# Efficacy of glaucoma drainage devices in uveitic glaucoma and a meta-analysis of the literature

**DOI:** 10.1007/s00417-018-4156-9

**Published:** 2018-10-11

**Authors:** Wishal D. Ramdas, Jan Pals, Aniki Rothova, Roger C. W. Wolfs

**Affiliations:** Department of Ophthalmology, Erasmus Medical Center, ‘s Gravendijkwal 230, 3000 CA Rotterdam, The Netherlands

**Keywords:** Glaucoma, Intraocular pressure, Glaucoma drainage device, Ahmed, Baerveldt, Uveitis

## Abstract

**Purpose:**

To assess the efficacy of glaucoma drainage devices (GDD) in uveitic glaucoma and non-uveitic glaucoma, and to perform a meta-analysis of previously published results to compare with our data.

**Methods:**

Retrospective case-control study, in which all eyes that underwent GDD surgery were included from 2015 onwards. Cases were defined as patients with uveitic glaucoma. Patients with non-uveitic glaucoma served as controls. To compare our results, a review of the literature was performed using PubMed database.

**Results:**

A total of 99 eyes were included (38 with uveitic glaucoma). The preoperative IOP was 25.9 ± 7.7 mmHg and 27.9 ± 9.6 mmHg for patients with and without uveitis (*p* = 0.277). No significant differences were found between patients with and without uveitis in the final IOP or reduction in IOP (44.9% vs. 42.8%, respectively). Within the first year after surgery, 13.2% of cases developed macular edema (vs. 6.6%; *p* = 0.267) and 15.8% a transient hypotony (vs. 8.2%; *p* = 0.242). A meta-analysis of 24 studies showed a postoperative weighted mean difference of − 17.8 mmHg and 2.2 lower number of IOP-lowering medications in uveitic glaucoma (compared to − 13.2 mmHg and 3.5 in the current study, respectively).

**Conclusion:**

GDD surgery in patients with uveitis has a similar effect on IOP as in patients without uveitis. The risks of developing macular edema and hypotony were slightly higher in patients with uveitis, but the results were not statistically significant. These findings are in line with previous reports, though data on the efficacy of GDD surgery and macular edema in uveitic glaucoma is scarce.

**Electronic supplementary material:**

The online version of this article (10.1007/s00417-018-4156-9) contains supplementary material, which is available to authorized users.

## Introduction

Glaucoma, a neurodegenerative eye disease, is the most common cause of irreversible blindness worldwide. Of all different forms of glaucoma, about 20–30% are caused by an underlying systemic or ophthalmic disorder: secondary glaucoma [1]. One of the most serious causes is uveitis, especially anterior uveitis [2]. The incidence of secondary glaucoma caused by uveitis (uveitic glaucoma) is reported to be 10–20% [3]. Patients with both glaucoma and uveitis have a high risk of severe visual impairment [2, 4]. Uveitic glaucoma presents a two-pronged problem: inflammatory damage to the trabecular meshwork and uvea (e.g., synechiae) coupled with steroid-induced increase in intraocular pressure (IOP). It is one of the most difficult forms of glaucoma to manage, because the ophthalmologist must simultaneously address inflammation and elevated IOP. Among the current surgical treatment options are trabeculectomy, deep sclerectomy, minimally invasive glaucoma surgery (MIGS; e.g., trabectome), cyclodestructive procedures, and several types of glaucoma drainage devices (GDD), with comparable efficacy [5–7]. However, the long-term role of trabeculectomy in patients with uveitis is compromised by young age, inflammation-induced fibrosis and conjunctival scarring, and scleral thinning, making trabeculectomy in many cases not the optimal choice. Moreover, uveitic glaucoma usually develops at a younger age (having a thick Tenon’s capsule and more robust healing response, often resulting in progressive subconjunctival fibrosis) than primary glaucoma. Cyclodestructive procedures are irreversible; hence, this treatment option is often reserved for eyes with a worse visual prognosis. Therefore, GDD are currently considered a better choice in the treatment of uveitic glaucoma. The most commonly used GDD are the valved Ahmed and the non-valved Baerveldt implant. Nonetheless, reports on the performance of these GDD in uveitis eyes are scarce. Therefore, we compared the efficacy and safety of the most common GDD (Ahmed and Baerveldt implant) in eyes with uveitic glaucoma to glaucoma eyes without uveitis. Next, we assessed the differences in complications after GDD surgery. Finally, we performed a review of the literature on these GDD (Ahmed FP7 and Baerveldt-350) in eyes with uveitic glaucoma and in eyes with other forms of glaucoma. Meta-analyses of the retrieved studies were performed and compared to our results.

## Methods

### Studies population

The first part of the study was designed as a retrospective study and the second part as a review with several meta-analyses (see further). For the retrospective study, we selected all patients that underwent at least one GDD surgery in the period from January 2015 to January 2018 at the Department of Ophthalmology of the Erasmus University Medical Center, Rotterdam, The Netherlands.

All patients underwent extensive ophthalmic examination at each visit. The medical records of all patients were reviewed, and clinically relevant data were entered in a database. Data of the last preoperative visit, postoperative follow-up data at 1 day, 1 week, 1 month (monthly till 6 months), 1 year, and their last visit were collected. The following data were recorded: age, gender, ethnicity, medical history, visual acuity, refraction, intraocular lens status, IOP, type of uveitis, complications of surgery, use of medication, date of surgery, operated eye, and type of GDD. Eyes that had previously failed GDD surgery were excluded.

### Surgical technique

All GDD surgeries were performed by the same surgeon (RW) using the following surgical technique. A limbal-based conjunctival flap was made in the superotemporal quadrant. An Ahmed FP7 (New World Medical, Rancho Cucamonga, CA, USA) or a Baerveldt BG101-350 (Abbott Medical Optics Inc., Santa Ana, CA, USA) GDD was placed 10 mm from the limbus, the Baerveldt with its wings underneath the lateral and superior rectus muscles. The plate was secured to the sclera with two nylon 9-0 sutures. The anterior chamber was entered using a 23 g knife after which the tube was inserted, with a preferred intraocular tube length of 3 mm. Next, the tube was sutured to the sclera with one nylon 9-0 suture and, in case of a Baerveldt GDD, tied off with a vicryl 6-0 suture. The extraocular part of the tube was patched with fascia lata (Tutoplast; Tutogen Medical, USA). Finally, the conjunctiva was closed with a running vicryl 8-0 suture.

The main reason for preferring an Ahmed GDD to a Baerveldt GDD were cases in which a quick postoperative IOP-lowering effect was required. At the time of surgery, there was no active inflammation. Postoperative topical medication (steroids and antibiotics) was similar for both GDD. Patients that were on preoperative immunosuppressive medications and/or antibiotic or antiviral systemic medications continued their use postoperatively at the discretion of the uveitis specialist (AR), who controlled the uveitis regularly during follow-up. If postoperative necessary glaucoma medication was added to reach target IOP.

### Assessment of main outcomes

The IOP was measured using Goldmann applanation tonometry (Haag-Streit, Köniz, Switzerland). The device had been calibrated according to manufacturer’s recommendations. The number of IOP-lowering medications was calculated by adding the number of different categories of medication. The categories were beta-blockers, prostaglandin-analogues, carbonic anhydrase inhibitors, alfa2-agonists, and oral acetazolamide. Fixed combinations of eye drops were calculated as two separate drugs. Patients with an unexplained decrease of visual acuity postoperatively underwent optical coherence tomography (OCT) of the macular region using Spectralis OCT (Heidelberg Engineering, Dossenheim, Germany) to detect underlying macular edema. Hypotony was defined as an IOP below 5 mmHg at two or more consecutive visits during the first year of follow-up.

### Search strategy and data extraction of studies

For the second part of the study, a review of the literature was performed by searching the PubMed database for studies on the Ahmed FP7 or Baerveldt-350 GDD up to April 2018. Studies had to assess the performance of at least one or both GDD on the IOP in either uveitic glaucoma or a mixture of different types of glaucoma. If a study used another type of Ahmed or Baerveldt GDD, the study was excluded from the meta-analyses. Extracted data included the first author, publication year, study design, sample size, diagnosis, type of GDD, follow-up duration, pre- and post-operative IOP, number of IOP-lowering medications pre- and postoperative, number of patients without any IOP-lowering medications at follow-up, number of secondary surgeries, and occurrence of macular edema or hypotony. From the research groups that published the results of the same study population more than once (e.g., 3-year follow-up and 5-year follow-up), only one study was included.

### Statistical analyses

Differences in general baseline characteristics of the patients were analyzed using independent *t* tests for continuous variables and chi-square tests for categorical variables. Within group analyses (e.g., between pre- and postoperative IOP) were analyzed using paired *t* tests.

Linear mixed models were applied to assess differences in IOP and IOP-lowering medication over time. Thus, two models were created in which one of these variables was fitted as the dependent variable with (fixed) visit as a factor, assuming a random effects intercept, and an unstructured correlation matrix. The models were adjusted for age, gender, type of GDD, and presence of uveitis. Follow-up time was calculated as the time between the date of surgery and the date of the last visit. If an eye required a second surgery during follow-up, follow-up was counted until the date of the second surgery to minimize confounding. All statistical analyses were performed using SPSS v22.0 for Windows (SPSS Inc., Chicago, IL, USA) and R statistical package version 2.11.1 for Mac (http://www.r-project.org). A *p* value of < 0.05 was considered statistically significant.

Data analysis of the literature review was performed using RevMan 5 for Windows (The Cochrane Collaboration, Oxford, UK). The mean and standard deviation were used to calculate the weighted mean differences (WMDs) with corresponding 95% confidence interval (CI). Heterogeneity was evaluated by calculating the *I*^2^-statistics and *p* values [8]. If heterogeneity was high (*I*^2^ > 50%), the studies that created the heterogeneity were excluded. This was done by sequentially omitting one study and reanalyzing the estimates of the remaining studies [9]. Results were pooled using the random-effects model in a meta-analysis. Using this method, the WMD of IOP across the retrieved studies was calculated for four groups: Ahmed GDD, Baerveldt GDD, both GDD in uveitic glaucoma, and in a mixture of different types of glaucoma. This approach was also applied to analyze the number of IOP-lowering medications.

## Results

A total of 99 eyes (86 patients) were included (38 with uveitic glaucoma). Of the patients with uveitic glaucoma, 44.7% had anterior uveitis, 2.6% had intermediate uveitis, 13.2% had posterior uveitis, and the remaining 39.4% had panuveitis. Table 1 shows the baseline characteristics of the study population and the underlying cause of the uveitis. Patients with uveitic glaucoma were significantly younger, more often female, and had a higher number of IOP-lowering medications compared to patients with non-uveitic glaucoma. Almost half of the patients had a history of intraocular surgery, most often cataract surgery (52.6% of the uveitic glaucoma eyes and 69.6% of the non-uveitic glaucoma eyes). The duration between the first episode of the uveitis and the first glaucoma surgery was median (interquartile range) 4.0 (2–7) years.

**Table 1:** Baseline preoperative characteristics of the study population presented as mean ± standard deviation unless stated otherwise

	Uveitic glaucoma (*N* = 38 eyes)	Non-uveitic glaucoma (*N* = 61 eyes)	*p* value
Age (years)^a^	44.4 ± 18.3	59.9 ± 19.3	< 0.001
Gender, female (*N*, %)^a^	24 (75.0)	28 (51.9)	0.034
Caucasian descent (*N*, %)^a^	22 (68.8)	40 (74.1)	0.595
IOP (mmHg)	25.9 ± 7.7	27.9 ± 9.6	0.277
Number of IOP-lowering Rx	4.24 ± 1.19	3.49 ± 1.30	0.005
Visual acuity	0.63 ± 0.38	0.50 ± 0.36	0.107
Spherical equivalent (D)^b^	− 1.46 ± 3.97	− 0.59 ± 2.99	0.436
Follow-up till censored (months)	13.0 ± 8.7	13.8 ± 9.3	0.686
Previous eye surgery (*N*, %)^c^	19 (50.0)	23 (37.7)	0.229
Uveitis diagnosis (*N*, %)			
Infectious		NA	
HSV	1 (2.6)		
VZV	1 (2.6)		
Rubella	2 (5.3)		
Associated systemic disease		NA	
Arthritis psoriatica	1 (2.6)		
Behcet	1 (2.6)		
HLA-B27 positive	1 (2.6)		
JIA	3 (7.9)		
Sarcoidosis	7 (18.4)		
Systemic sclerosis	1 (2.6)		
Clinical ocular syndromes		NA	
Birdshot chorioretinopathy	2 (5.3)		
FHUS (negative for viruses)	1 (2.6)		
Idiopathic		NA	
Quantiferron positive	5 (13.2)		
Quantiferron negative/e.c.i.	11 (28.9)		

*NA* not applicable/available, *IOP* intraocular pressure, *FHUS* Fuchs heterochromic uveitis syndrome, *JIA* juvenile idiopathic arthritis, *TBC* tuberculosis, *HSV* herpes simplex virus, *VZV* varicella zoster virus

^a^Data per patient instead of per eye

^b^Excluding eyes that underwent cataract surgery

^c^Including surgery for glaucoma (*N* = 8), cataract (*N* = 26), trans pars plana vitrectomy (*N* = 2), cornea transplants (*N* = 3), and other (*N* = 3)

**Fig. 1:**
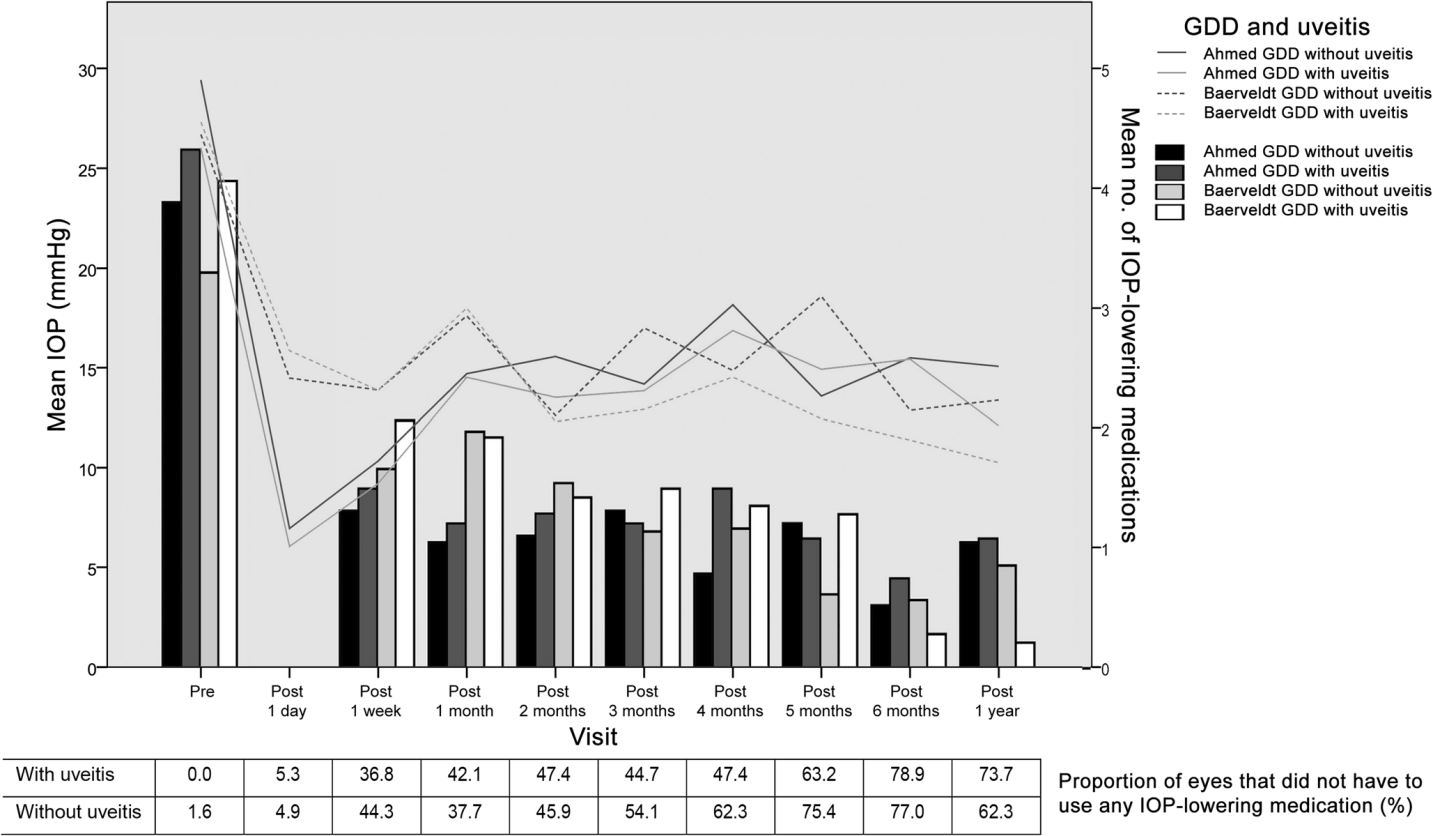
Mean intraocular pressure (IOP; lines) and number of IOP-lowering medications (bars) during follow-up for non-uveitic glaucoma treated with the Ahmed FP7 glaucoma drainage device (GDD; solid black line; black bar), non-uveitic glaucoma treated with Baerveldt-350 GDD (dotted black line; light gray bar), uveitic glaucoma treated with Ahmed FP7 GDD (solid gray line; black bar), and uveitic glaucoma treated with Baerveldt-350 GDD (dotted gray line; white bar)

Figure 1 displays the IOP levels (separately for Ahmed GDD and Baerveldt GDD) and number of IOP-lowering medications for the whole study population during follow-up. Nine eyes underwent a second surgery within the first year after the GDD surgery. Of these, one had uveitic glaucoma and underwent regular cataract extraction. The remaining (eight) had non-uveitic glaucoma of which one underwent regular trans pars plana vitrectomy, one developed neovascular glaucoma for which cyclocryotherapy was applied, and six required GDD-related surgery because of tube malposition (*N* = 2), tube protrusion through the conjunctiva (*N* = 1), tube removal (persistent hypotony; *N* = 2), and tube flushing (*N* = 1). As we included two different GDD (Ahmed and Baerveldt), we first assessed whether there were differences in the results between both GDD. None of the in Table 1 presented baseline characteristics were significantly different between both GDD, except for the preoperative number of IOP-lowering medications (4.14 ± 1.36 and 3.50 ± 1.21 [*p* = 0.015] for the Ahmed GDD and Baerveldt GDD, respectively). The IOP in the Ahmed GDD group decreased from 27.5 ± 9.1 mmHg preoperative to 14.5 ± 4.5 mmHg (*p* < 0.001; *N* = 43) at the last follow-up, and in the Baerveldt GDD group from 26.9 ± 8.9 mmHg to 12.1 ± 3.8 mmHg (*p* < 0.001; *N* = 56). Figure 1 also shows that in the very early postoperative phase, the IOP was significantly lower with an Ahmed GDD compared to a Baerveldt GDD; however, at 6 months, this changed to a significantly higher IOP (*p* = 0.031). Also at the last visit (> 1 year postoperative), eyes with an Ahmed GDD had still a significantly higher IOP compared to the Baerveldt GDD (*p* = 0.005). The only significant difference in number of IOP-lowering medications during follow-up was at 1 month postoperative (1.14 ± 1.25 and 1.96 ± 1.74 [*p* = 0.010] for the Ahmed GDD and Baerveldt GDD, respectively). Because of the comparable performance of both GDD on the IOP, we combined both GDD into one group with uveitic glaucoma and one with non-uveitic glaucoma. As expected, the IOP decreased significantly after GDD surgery in eyes with uveitic glaucoma from 25.9 ± 7.7 to 12.7 ± 4.4 mmHg (44.9% decrease; *p* < 0.001), and in eyes with non-uveitic glaucoma from 27.9 ± 9.6 to 13.3 ± 4.2 mmHg (42.8% decrease; *p* < 0.001). The postoperative decrease in IOP in eyes with uveitic glaucoma did not differ with the decrease in IOP in eyes with non-uveitic glaucoma (*p* = 0.729). If we take the IOP of the whole follow-up period into account, the linear mixed model showed that age and uveitis were significantly associated with IOP during follow-up. The presence of uveitis was associated with a 1.93 mmHg lower IOP over time compared to eyes without uveitis (*p* = 0.008). The number of IOP-lowering medications was not significantly different between eyes with and without uveitis during follow-up. Approximately 70% of eyes did not have to use any IOP-lowering medication after 6 months of GDD surgery (Fig. 1). At the last follow-up, the mean ± standard deviation number of IOP-lowering medications was similar for the uveitic and non-uveitic glaucoma eyes (0.76 ± 1.34 and 0.92 ± 1.33, respectively; *p* = 0.576).

Macular edema developed in 13.2% of eyes with uveitic glaucoma (vs. 6.6%; *p* = 0.267) and hypotony in 15.8% (vs. 8.2%; *p* = 0.242). In both groups, the visual acuity and spherical equivalent did not change significantly during follow-up. Also, at the end of follow-up, the visual acuity did not differ between eyes with uveitic and non-uveitic glaucoma (*p* = 0.317).

The literature search yielded a total of 24 studies that assessed the performance of the Ahmed FP7 or Baerveldt-350 GDD (Table S1) [10–33]. Most studies analyzed a mixture of different types of glaucoma. In uveitic glaucoma, 6–65% of eyes did not have to use any IOP-lowering medication postoperative (compared to 3–83% in the glaucoma mixture studies), postoperative macular edema developed in 2–26% (compared to 2–4% in the glaucoma mixture studies), and hypotony was reported to be present in 0–36% (compared to 1–37% in the glaucoma mixture studies). However, postoperative macular edema was assessed in only six studies.

**Fig. 2:**
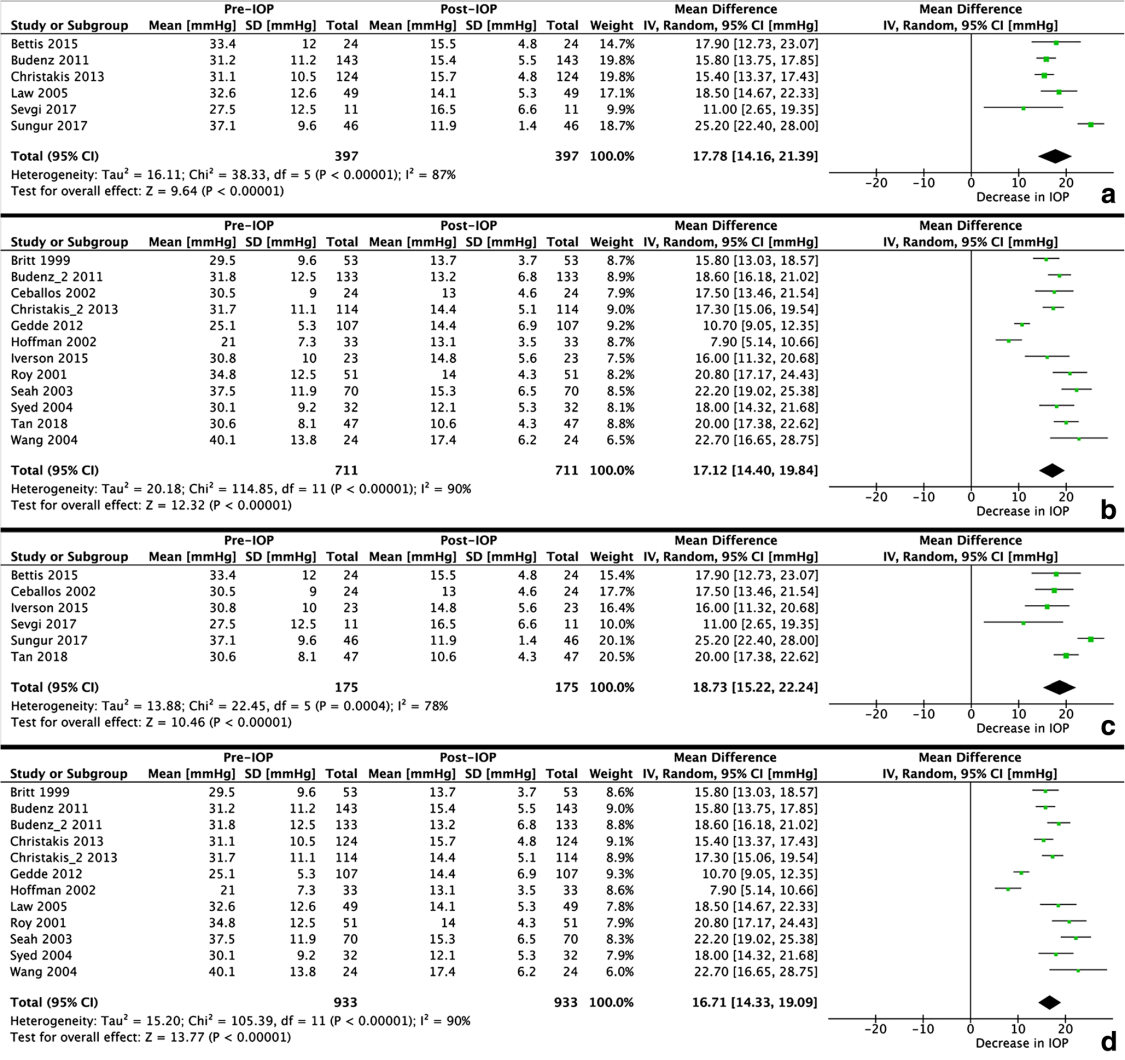
Meta-analyses for the postoperative change in IOP after the Ahmed FP7 (A), Baerveldt-350 (B), both GDD in uveitic glaucoma (C), and both GDD in a mixture of different types of glaucoma (D)

**Fig. 3:**
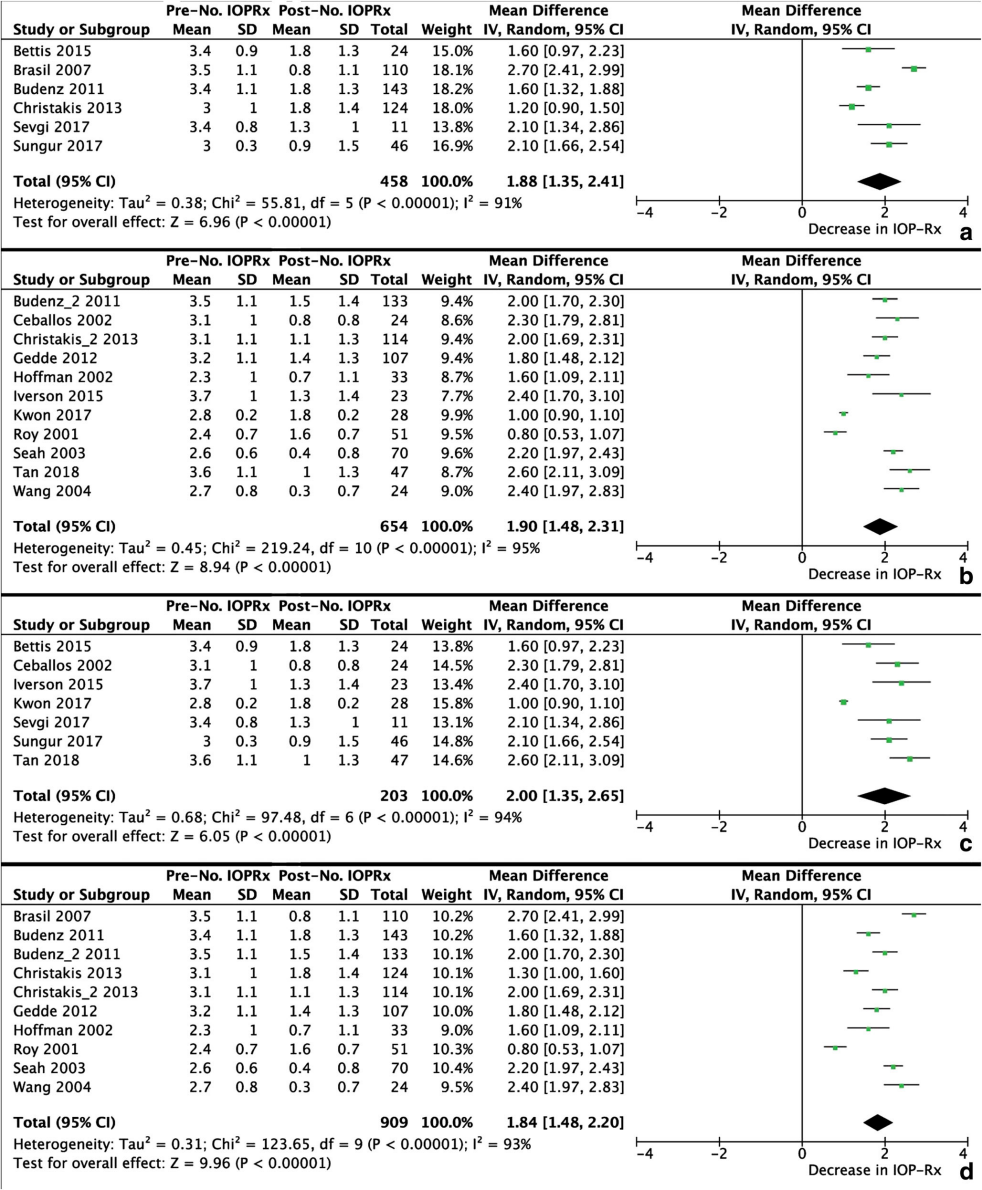
Meta-analyses for the postoperative change in number of IOP-lowering medications after the Ahmed FP7 (A), Baerveldt-350 (B), both GDD in uveitic glaucoma (C), and both GDD in a mixture of different types of glaucoma (D)

Of the 24 retrieved studies, 10 were excluded from the meta-analyses for IOP and 11 were excluded from the meta-analyses for IOP-lowering medication because of missing data. Figures 2 and 3 present the results of the meta-analyses for the performance of the Ahmed FP7, the Baerveldt-350, both GDD in uveitic glaucoma, and both GDD in a mixture of different types of glaucoma, on the IOP and IOP-lowering medication, respectively. In all analyses, substantial heterogeneity was present. However, after omitting the studies that contributed to the heterogeneity, the results did not change significantly. The WMD [95% CI] (*I*^2^) for IOP (in mmHg) were 15.96 [14.67–17.25] (0%), 18.69 [17.32–20.07] (44%), 17.76 [15.43–20.08] (29%), and 17.77 [16.42–19.11] (38%), respectively. For IOP-lowering medication, the WMD [95% CI] (*I*^2^) were 1.80 [1.51–2.09] (34%), 2.11 [1.93–2.29] (49%), 2.21 [1.94–2.48] (26%), and 2.02 [1.83–2.21] (48%), respectively.

In our study population, eyes with uveitic glaucoma had a mean difference in IOP of 13.18 mmHg and in IOP-lowering medications of 3.47.

## Discussion

The efficacy of GDD in uveitic vs. non-uveitic glaucoma in a tertiary center was presented and compared with the literature by performing several meta-analyses. The results of GDD surgery in eyes with uveitic glaucoma are similar to those of eyes with non-uveitic glaucoma. Both groups showed a decrease in IOP of ~ 42% after GDD surgery. Although eyes with uveitic glaucoma developed more often macular edema and hypotony compared to eyes with non-uveitic glaucoma, the difference was not statistically significant.

There are not many studies that compared the performance of GDD in patients with and without uveitic glaucoma. Most studies analyzed a mixture of different types of glaucoma making it hard to compare our results. Our literature search identified four prospective randomized controlled trials on either the Ahmed FP7 or Baerveldt-350 GDD. Nonetheless, all studies on the efficacy of GDD in uveitic glaucoma were retrospective studies. None of the 24 identified studies assessed the difference of GDD between patients with and without uveitis, except for one [14]. Nevertheless, that study only included the Ahmed FP7 and not the Baerveldt-350. Furthermore, their sample size (*N* = 15) was very small. Our finding that the decrease in IOP after GDD surgery is similar in eyes with and without uveitis is in agreement with their results [14]. In contrast to the current study, they reported an increase in number of IOP-lowering medications 1-month postoperatively. This increase was higher in open-angle glaucoma eyes compared to uveitic glaucoma eyes, and IOP-lowering medications became stable at 2 years postoperative [14]. However, the number of postoperative IOP-lowering medications was highest in their study (2.6 in uveitic glaucoma and 2.8 in non-uveitic glaucoma) among all retrieved studies. It should be noted that in the current study, the mean IOP at baseline was lower compared to other studies, but this might be related to the higher number of IOP-lowering medications used by our patients (Fig. 3C, D). The non-significant difference in IOP between uveitic glaucoma and other forms of glaucoma may also be observed from the results of the meta-analyses (Fig. 2C, D).

Our proportion of eyes requiring secondary surgery related to the GDD (6.1%) is similar to other studies reporting 0–13% (Table S1). Due to the retrospective design of most studies, the development of macular edema may have been underreported. Nevertheless, according to our results and the previous reports (Table S1), it is likely that the risk of developing macular edema after GDD surgery is higher in uveitic glaucoma compared to non-uveitic glaucoma. The development of (transient) hypotony in our study (15.8% in uveitic glaucoma and 8.2% in non-uveitic glaucoma) is in agreement with others; however, the reported range in other studies is extremely wide: 0–37.1% (Table S1).

One of the strengths of the current study is that the performance of the GDD did not depend on the great variability in how glaucoma specialists perform GDD surgery, because all surgeries were performed by the same surgeon using the same technique each time. Also, the indication for surgery was set by the same surgeon. To minimize the effect of different GDD on the IOP, only two types of GDD were included. Furthermore, the IOP was measured at each visit using the same method.

Due to the retrospective design of the current study, there are several limitations. First, the follow-up of our study was relatively short. Nevertheless, according to the tube versus trabeculectomy study, the IOP of the Baerveldt GDD did not change between 1-year follow-up and 5-year follow-up [31]. Moreover, the Ahmed Baerveldt Comparison study and Ahmed versus Baerveldt study did not show significant differences in IOP after 6 months [28, 30]. The study that compared the Ahmed GDD efficacy in uveitic glaucoma to open-angle glaucoma patients had a longer follow-up than the current study, but their results on IOP did not change significantly after 3 months of follow-up [14]. Second, our differences between Ahmed GDD and Baerveldt GDD may suffer from selection bias, because the surgeon may have a preference for a certain GDD in specific situations. For example, the higher drop in IOP in the first week after Ahmed GDD surgery (compared to Baerveldt GDD; Fig. 1) may result from this selection bias next to the valve mechanism. However, the significantly higher IOP after Ahmed GDD surgery compared to Baerveldt GDD at the end of follow-up is in agreement with two other studies that compared the Ahmed FP7 with the Baerveldt-350 GDD [28, 30]. Moreover, the preoperative IOP level did not differ between Ahmed GDD and Baerveldt GDD (*p* = 0.660; Fig. 1), suggesting that selection bias based on the preoperative IOP played a minor role. Regarding the meta-analyses, if different types of non-uveitic glaucoma would result in different outcomes of GDD surgery, caution should be taken when interpreting the studies including a mixture of types of glaucoma. The heterogeneity between studies may also be explained by the many different factors that may affect postoperative GDD results (e.g., experience of surgeon, surgical technique, follow-up duration, IOP measurement methods, method used to calculate number of IOP-lowering medications, severity of glaucoma, etc.).

The risk of developing secondary glaucoma due to uveitis depends on various factors such as the age, ethnicity, duration of inflammation, the clinical manifestations of uveitis, and its therapy. High risk for glaucoma development was especially noted for viral causes and anterior location of uveitis [34]. Our results are consistent with these findings, as the majority of our patients exhibited anterior segment involvement of uveitis.

In conclusion, both the Ahmed FP7 and the Baerveldt-350 GDD are effective in lowering IOP in uveitic glaucoma. Their performance in terms of IOP and IOP-lowering medications is similar to eyes with non-uveitic glaucoma. Also, the risk of developing macular edema or hypotony after GDD was not different in uveitic glaucoma compared to non-uveitic glaucoma. Unfortunately, prospective studies that assessed the clinical manifestations of uveitis in relation to GDD surgery are currently lacking. Future prospective randomized controlled trials on uveitic glaucoma are required to verify the current results.

## Electronic supplementary material

Table S1 (DOCX 42.9 kb)
