# Supplementary material for: Efficacy of glaucoma drainage devices in uveitic glaucoma and a meta-analysis of the literature
Source: Graefes Arch Clin Exp Ophthalmol. 2018 Oct 11;257(1):143–51. doi: 10.1007/s00417-018-4156-9 (PMC6323086; doi:10.1007/s00417-018-4156-9)
Supplement: Supplementary file 1 — (DOCX 42.9 kb) [file 417_2018_4156_MOESM1_ESM.docx]

Table S1. Overview of other studies on the performance of Ahmed FP7 and/or Baerveldt-350 GDD in alphabetical order. Data of other GDD or surgical alternatives that were assessed in the studies are not shown.

|  | Study |  | Design | N (eyes) per group | Diagnosis | Type of GDD | Follow-up (mean±sd) | No IOP-Rx, % | Secondary surgeries, % | Hypotony, % | Macular edema, % | Comparison |
| --- | --- | --- | --- | --- | --- | --- | --- | --- | --- | --- | --- | --- |
| 1 | Aung, et al. 1998 | R | case series | 24 | Glaucoma | B | 13.4 | 21 |  |  |  | B & Molteno |
| 2 | Bettis, et al. 2015 | R | case-control | 24 | UG | A | 23.9±12.2 | 41.2 |  | 4.2 |  | A vs TE |
| 3 | Brasil, et al. 2007 | R | case-control | 110 | Glaucoma | A | 12 |  | 9.1 | 33.6 |  | A S2 vs FP7 |
| 4 | Britt, et al. 1999 | P | RCT | 53 | Glaucoma | B | 37 |  |  |  |  | B 350 vs 500 |
| 5 | Budenz, et al. 2011 | P | RCT | 143 | Glaucoma | A | 12 | 23 | 5 | 3 | 6 | A FP7 vs B 350 |
|  |  |  |  | 133 | Glaucoma | B | 12 | 36 | 13 | 2 | 2 |  |
| 6 | Ceballos, et al. 2002 | R | case series | 24 | UG | B | 20.8 | 58.3 |  | 12.5 | 12.5 | B 250 & 350 & 425 |
| 7 | Christakis, et al. 2013 | P | RCT | 124 | Glaucoma | A | 36 | 25 | 38* | 11 |  | A FP7 vs B 350 |
|  |  |  |  | 114 | Glaucoma | B | 36 | 50 | 50* | 13 |  |  |
| 8 | Gedde, et al. 2012 | P | RCT | 107 | Glaucoma | B | 60 | 25 | 9 | 2.8 |  | B 350 vs TE |
| 9 | Hoffman, et al. 2002 | R | case series | 33 | Glaucoma | CE+B | 15,4±9,4 |  |  |  |  | CE combined with B |
| 10 | Iverson, et al. 2015 | R | case-control | 23 | UG | B | 39±19 |  | 0 | 17.4 | 26.1 | B 250 & 350 vs TE |
| 11 | Kwon, et al. 2017 | R | case-control | 28 | UG | B/Molteno | 31,2±22,9 | 28 |  | 36 |  | B 250 & 350 & Molteno |
| 12 | Law, et al. 2005 | R | case-control | 49 | Glaucoma | A | 12 |  |  | 2.0 |  | A S2 vs FP7 |
| 13 | Mackenzie, et al. 2006 | R | case-control | 48 | Glaucoma | A | 20,1±8,8 |  |  |  |  | A S2 vs FP7 |
| 14 | Rachmiel, et al. 2008 | R | case-control | 15 | UG | A | 31,7±17,7 |  |  | 26.2 |  | A FP7 in UG vs OAG |
|  |  |  |  | 53 | OAG | A | 32,4±19,7 |  |  | 9.4 |  |  |
| 15 | Roy, et al. 2001 | R | case series | 51 | Glaucoma | B | 37,6±18,8 | 29 |  | 15.7 |  | B 250 & 350 |
| 16 | Seah, et al. 2003 | R | case-control | 70 | Glaucoma | B | 32,2±13,7 | 57 |  | 7 |  | B 250 vs 350 |
| 17 | Sevgi, et al. 2017 | R | case-control | 11 | UG | A | 12 |  | 9.1 | 0 |  | A vs. A+fluocinolone implant |
| 18 | Siegner, et al. 1995 | R | case-control | 55 | Glaucoma | B | 13,6±0,9 |  |  | 38 | 1.9 | B 200 vs 250 vs 350 vs 500 |
| 19 | Smith, et al. 1995 | R | case-control | 18 | Glaucoma | B | 10,1 | 24 |  | 11.1 |  | B vs Molteno |
| 20 | Sungur, et al. 2017 | R | case series | 46 | UG | A | 51,9±23,1 | 6.5 | 2 | 26.1 |  | A in UG |
| 21 | Syed, et al. 2004 | R | case-control | 32 | Glaucoma | A | 12 | 12.5 | 3.1 | 34.4 |  | A S2 vs B 350 |
|  |  |  |  | 32 | Glaucoma | B | 12 | 3 |  | 37.5 |  | A S2 vs B 350 |
| 22 | Tan, et al. 2018 | R | case series | 47 | UG | B | 63,6±43,1 | 65 |  | 11 | 2 | B 250 & 350 in UG |
| 23 | Tsai, et al. 2006 | R | case-control | 70 | Glaucoma | B | 48 | 64 | 6 | 1 |  | A S2vs B 250 & 350 |
| 24 | Wang, et al. 2004 | R | case-control | 24 | Glaucoma | B | 22,8±8,7 | 83.3 |  | 37.5 | 4.2 | A S2vs B 350 |

GDD = glaucoma drainage device; IOP-Rx = intraocular pressure-lowering medications; sd = standard deviation; P = prospective; R = retrospective; RCT = randomized controlled trial; Glaucoma = mixture of different types of glaucoma; UG = uveitic glaucoma; OAG = open-angle glaucoma; CE = cataract extraction; A = Ahmed GDD; B = Baerveldt GDD; TE = trabeculectomy; * = including regular surgeries
